# Enterprise application integration task-based execution model experimental dataset

**DOI:** 10.1016/j.dib.2022.108101

**Published:** 2022-03-26

**Authors:** Rafael Z. Frantz, Raquel M. Pillat, Fabricia Roos-Frantz, Sandro Sawicki, Fábio P. Basso

**Affiliations:** a Unijuí University, Ijuí/RS, Brazil; b Federal University of Rio de Janeiro, Rio de Janeiro/RJ, Brazil; c Federal University of Pampa, Alegrete/RS, Brazil

**Keywords:** Enterprise application integration, Integration platform, Integration framework, Performance analysis, Makespan, Workflow execution

## Abstract

This data article provides raw log files captured from automated computational experiments with an application integration platform that implements the task-based execution model. The code of a well-known enterprise application integration process was modified to record these data while running the experiments. The dataset described in this article is related to the research article entitled “On the Analysis of Makespan and Performance of the Task-based Execution Model for Enterprise Application Integration Platforms: an Empirical Study” of Frantz et al. (2021). The dataset contains two types of CSV files in text format presenting: (1) data of makespan related to experiment executions and (2) data regarding the number of messages and work-units processed during each experiment. Scientific community who works on workflow and integration process execution can benefit from these data to analyse and understand the behaviour of the task-based execution model, obtain insights to improve this model, as well as perform comparisons with other types of execution models or platforms.

## Specifications Table

**Table:** 

Subject	Information Systems; Computer Science Applications.
Specific subject area	Enterprise application integration.
Type of data	Log files (text CSV)
How data were acquired	Through automated computational experiments in which an enterprise application integration software produces data for each executed experiment.
Data format	Raw
Parameters for data collection	Experiments were carried out on a machine equipped with 16 processors Intel Xeon CPU E5-4610 V4, 1.8 GHz, 32GB of RAM, and operating system Windows Server 2016 Datacenter 64-bits. Java SE version 8.0 update 152 was installed and Guaraná integration platform version 1.4. No other software was installed in this machine, and it was disconnected from the Internet.
Description of data collection	The code of a well-known enterprise application integration process, executed by an application integration platform, was modified to record relevant data while running computational experiments. Input data for experiments were synthetically generated and injected in the integration process. Output data were automatically collected and stored in log files.
Data source location	Institution: Unijuí University City/Town/Region: Ijuí/RS Country: Brazil
Data accessibility	Data is hosted and made publicly available at GitHub. Repository name: GitHub Data identification number: 10.5281/zenodo.4758545 (DOI), can be cited according to reference [4] Direct URL to data: https://github.com/gca-research-group/task-based-dataset
Related research article	Author's name: Rafael Z. Frantz, Sandro Sawicki, Fabricia Roos-Frantz, Fábio P. Basso, Benjamim Zucoloto, Raquel M. Pillat. Title: On the Analysis of Makespan and Performance of the Task-based Execution Model for Enterprise Application Integration Platforms: an Empirical Study [1]. Journal: Software - Practice and Experience Article DOI: 10.1002/SPE.3085

## Value of the Data

• Execution models available in the literature and adopted by runtime systems of integration platforms must be revised and probably reengineered now considering this kind of systems are increasingly having to deal with large volumes of data, which is a different scenario from the past when they were conceived. In this sense, the presented dataset captures the behaviour of these systems when large volumes of input data are introduced.
• Scientific community who works on workflow and integration process execution can benefit from these data.
• These data can be used to analyse and understand the behaviour of the task-based execution model and provide insights to improve this model.
• These data may also be used to perform comparative analysis studies, in which data related to the task-based execution model (reported in this article) would be compared to data captured from the process-based execution model. Still, the presented data may be used to investigate similarities and/or differences between the integration platform applied in this study to other existing ones.
• Threads used by the runtime systems to execute integration processes do not correspond to kernel or physical threads in the hardware, i.e., they are virtual user threads. This dataset can also be used to study the actual improvement in performance by increasing the number of virtual user threads despite the limited number of kernel or physical threads.

## 1 Data

The dataset provided with this article contains execution logs of a well-known application integration process, the Coffee Shop process [2], enacted with a task-based integration platform [3]. The dataset has 2 different types of *txt* files, which are described in Table 1. Variable IR, which composes the name of these files, represents an experiment variable. In this article, we consider that each experiment corresponds to 25 executions of the integration process considering the same combination of specific values for variables. Every experiment was repeated 25 times due to the statistical reasons explained in the Methods section and we have considered a fixed running time of 60 seconds in each repetition.

**Table 1:** Overview of files in the dataset provided with this article. Legend: IR = message input rate.

File	Description
makespan-[IR].txt	Records raw data for the makespan. Makespan is a well-known metric that measures the time a message takes to complete its execution in the workflow of an integration process.
	Every file with this name contains data for experiments considering a single input rate indicated by the variable IR and the whole set of 50 threads.
messages-workunits*-*[IR].txt	Records raw data regarding the number of messages and work-units processed. The former corresponds to the total number of messages received by the integration process during the 60-second time-frame interval of the experiment, whereas the latter corresponds to the total number of workflow tasks processed in the integration process during the 60-second time-frame interval of the experiment. A work-unit represents the execution of a message in each workflow task. The workflow of the Coffee Shop integration process, used in our experiments, is composed of 20 tasks in which a message must be executed. Therefore, every inbound message gives place to 20 work-units.
	Every file with this name contains data for experiments considering a single input rate indicated by the variable IR and the whole set of 50 threads.

Variable IR (**I**nput **R**ate) corresponds to the message input rate, i.e., the number of messages that have been injected into the integration process per second. Fifty-seven (57) different input rates were considered in the range of 1 to 10,000 inbound messages per second (msg/s). Moreover, we have also varied in the experiments the number of threads available in the global pool of the integration platform's run-time system from 1 to 50 threads. Therefore, the full dataset contains 2,850 experiments varying values for these variables (57 input rates x 50 numbers of threads).

**Fig. 1:**
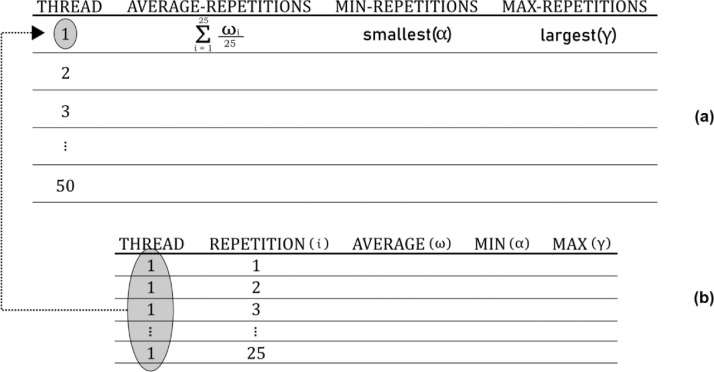
Layout of dataset in files *makespan-[IR].txt*

Data for the makespan follow the layout presented in Fig. 1. At the top of this figure, layout (a) shows the computed data for every number of threads (1 to 50), whereas layout (b) in the bottom shows computed data in every 25 repetitions of an experiment using the same number of threads. In the file, this bunch of data for 25 repetitions of an experiment in layout (b) is repeated 50 times, i.e., for each number of threads considered. In each repetition, column “average (*ω*)” records the average makespan computed for those messages that were completely executed in the integration process workflow, column “min (*α*)” records the smallest makespan and column “max (*γ*)” the largest makespan in every repetition. Data in layout (a) can be seen as a summary of data in layout (b), since column “average-repetitions” records the average makespan considering average values (*ω*) of every 25 repetitions; column “min-repetitions” records the smallest makespan found in 25 repetitions; and column “max-repetitions” records the largest makespan found in 25 repetitions.

**Fig. 2:**
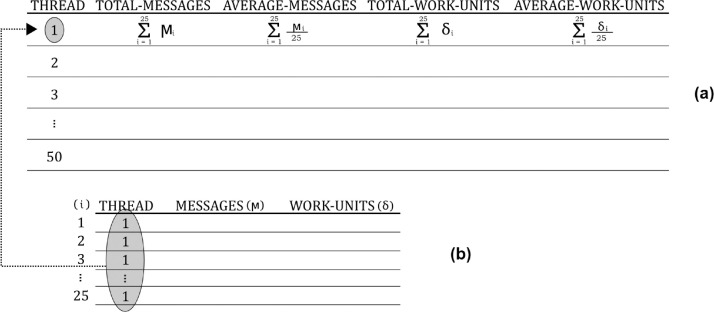
Layout of dataset in files *messages-workunits-[IR].txt*

Data regarding messages and work-units processed follow the layout presented in Fig. 2. At the top of this figure, layout (a) shows the computed data for every number of threads (1 to 50), whereas layout (b) in the bottom shows the computed data in every 25 repetitions of an experiment using the same number of threads. In the file, this bunch of data for 25 repetitions of an experiment in layout (b) is repeated 50 times, i.e., for each number of threads considered. In each repetition, column “messages (*μ*)” records the number of messages that were completely executed in the integration process workflow and column “work-units (*δ*)” records the number of work-units executed in every repetition. Data in layout (a) can be seen as a summary of data in layout (b), since column “total-messages” records the sum of processed messages (*μ*) () every 25 repetitions; column “average-messages” records the average number of processed messages that were completely executed in the integration process workflow in each repetition; column “total-work-units” records the sum of processed work-units (*δ*) every 25 repetitions; and column “average-work-units” records the average number of processed work-units in each repetition.

## 2 Experimental Design and Materials

Dataset accompanying this article has been gathered from an experimental study aiming to evaluate the task-based execution model in the context of enterprise application integration by means of an actual integration platform that implements this model [1]. The experimental design was created to analyse the impact of the volume of inbound messages arriving in the integration process and the number of threads available in the global pool of the integration platform on makespan and performance. Makespan measures the time a message takes to complete its execution in the workflow of an integration process whereas performance, in this context, is related to the number of messages processed per second in an integration process. In this study, we have used the Guaraná integration platform [3], which is open-source and implements the task-based execution model. The following summarizes other issues related to the experimental design. A complete description of this experimental study and performed analyses can be found in our related research article [1].

***Experimental subject***: we have chosen the Coffee Shop integration process [2] to execute our experiments, since it has become the *de facto* standard to demonstrate and evaluate integration platforms from a practical point of view.

***Environment***: Experiments have been executed on a machine equipped with 16 processors IntelXeon CPU E5-4610 V4, 1.8 GHz, 32GB of RAM, and operating system WindowsServer 2016 Datacenter 64-bits. Guaraná integration platform version 1.4 was also installed as well as Java SE version 8.0 update 152.

***Experimental variables:*** As part of the experimental design, we also defined dependent and independent variables. *Running time* (of each experiment), *message input rate*, and *number of threads* are the independent variables of the study. On the other hand, *makespan* and *number of messages processed* are variables the experimental study intended to evaluate and, therefore, they are dependent measures from the first ones.

**Code files**: The source code used to generate the experimental data is available at GitHub (https://github.com/gca-research-group/task-based-experiment-project) and can be cited according to reference [5].

## 3 Methods

In statistics, when an experiment is repeated a large number of times with the same data, as the number of repetitions increases, the sample mean of experiment variables approaches the population mean. Although the number of repetitions may vary depending on the “stability” of results, in this experimental study, the population mean was achieved mostly with 25 repetitions. Thus, each experiment was repeated 25 times and results, excluding outliers, were averaged to diminish the effects of unpredictable events in the operating system. As first code executions by the Java virtual machine tend to be slower and a warmup is recommended to let the virtual machine eventually perform code optimisation, early 100 experiments were performed twice to drop first executions. Moreover, we introduced a 60-second delay between each two experiments. In this way, the garbage collector of the Java virtual machine is called to remove unused objects and release memory space.

By definition, messages that flow through the integration process are formed by a header and a body. The header contains predefined and custom properties. The body holds the payload data. In every experiment, the message body holds an actual document in XML format and the size of a message being processed by an integration process varies, as it is modified and transformed throughout the workflow. Thereby, we computed the average size of messages belonging to a same correlation processed in an integration process. The result is an average message size of 1376.40 bytes.

We modified the source code of the Coffee Shop integration process to add a custom property to the header of every inbound message. This alteration aimed to store the time the message enters to the workflow of the integration process. The makespan of the integration process for a given message corresponds to the difference between the time it reached the end of the workflow and the time it has entered. We compute the makespan for every message that has completed the workflow in the experiment, and, afterwards, we compute the mean value. We also modified the source code of the process to include a counter for the number of messages processed, i.e., which have completed the workflow in every experiment. Messages were synthetically generated and injected into the integration process following input rates in every experiment. Data regarding the makespan and number of messages processed during the execution of every experiment and its repetitions were automatically collected and stored in a log file to be later processed and analysed.

## Declaration of Competing Interest

None.

## Data Availability

Task based experiment project (Original data). Java source code used to generate the dataset (Original data). Dataset generated in the task-based experiment (Original data). Enterprise Application Integration Task-Based Execution Model Experimental Dataset (Original data) (GitHub).
